# Biological Control of Celery Powdery Mildew Disease Caused by *Erysiphe heraclei* DC In Vitro and In Vivo Conditions

**DOI:** 10.3390/plants10112342

**Published:** 2021-10-29

**Authors:** Hamada F. A. Ahmed, Mahmoud F. Seleiman, Adel M. Al-Saif, Maha A. Alshiekheid, Martin L. Battaglia, Ragab S. Taha

**Affiliations:** 1 Department of Ornamental, Medicinal and Aromatic Plant Diseases, Plant Pathology Research Institute, Agricultural Research Center (ARC), Giza P.O. Box 12619, Egypt; dr.hamada.faa@gmail.com; 2 Department of Plant Production, College of Food and Agriculture Sciences, King Saud University, P.O. Box 2460, Riyadh 11451, Saudi Arabia; adelsaif@ksu.edu.sa; 3 Department of Botany and Microbiology, College of Science, King Saud University, Riyadh 11451, Saudi Arabia; malsheikh@ksu.edu.sa; 4 Department of Animal Sciences, Cornell University, Ithaca, NY 14850, USA; mlb487@cornell.edu; 5 Botany Department, Faculty of Agriculture, Beni-Suef University, Beni Suef 62521, Egypt; ragab.salama@agr.bsu.edu.eg

**Keywords:** biological control, powdery mildew, *Apium graveolens*, *Erysiphe heraclei*, conidia germination, leaf pigments, defense-related enzymes

## Abstract

The present study aimed to investigate the potentiality of certain biocontrol agents, namely *Bacillus subtilis*, *B. pumilus*, *B. megaterium*, *Pseudomonas fluorescens*, *Serratia marcescens*, *Trichoderma album*, *T. harzianum* and *T. viride*, as well as the synthetic fungicide difenoconazole to control celery powdery mildew caused by *Erysiphe heraclei* DC, in vitro (against conidia germination and germ tube length of *E. heraclei*) and in vivo (against disease severity and AUDPC). In vitro, it was found that the antifungal activity of the tested biocontrol agents significantly reduced the germination percentage of the conidia and germ tube length of the pathogen. The reduction in conidia germination ranged between 88.2% and 59.6% as a result of the treatment with *B. subtilis* and *T. album*, respectively compared with 97.1% by the synthetic fungicide difenoconazole. Moreover, the fungicide achieved the highest reduction in germ tube length (92.5%) followed by *B. megaterium* (82.0%), while *T. album* was the least effective (62.8%). Spraying celery plants with the tested biocontrol agents in the greenhouse significantly reduced powdery mildew severity, as well as the area under the disease progress curve (AUDPC), after 7, 14, 21 and 28 days of application. In this regard, *B. subtilis* was the most efficient followed by *B. pumilus*, *S. marcescens* and *B. megaterium*, with 80.1, 74.4, 73.2 and 70.5% reductions in disease severity, respectively. In AUDPC, reductions of those microorganisms were 285.3, 380.9, 396.7 and 431.8, respectively, compared to 1539.1 in the control treatment. On the other hand, the fungicide difenoconazole achieved maximum efficacy in reducing disease severity (84.7%) and lowest AUDPC (219.3) compared to the other treatments. In the field, all the applied biocontrol agents showed high efficiency in suppressing powdery mildew on celery plants, with a significant improvement in growth and yield characteristics. In addition, they caused an increase in the concentration of leaf pigments, and the activities of defense-related enzymes such as peroxidase (PO) and polyphenol oxidase (PPO) and total phenol content (TPC). In conclusion, the results showed the possibility of using tested biocontrol agents as eco-friendly alternatives to protect celery plants against powdery mildew.

## 1. Introduction

Celery (*Apium graveolens* L. var. *dulce* (Mill.) Pers.) is an annual or biennial herbaceous plant in the family Apiaceae, originating in the Mediterranean and the Middle East [1], and grows widely in tropical and subtropical regions of Africa, Asia, Central Europe and western India [2]. It is commonly cultivated for its green leaves, bulbous roots, seeds (fruits) and petioles [3], that are used for nutritional and medicinal purposes [4]. The whole plant exhibits an aromatic flavor, and its leaf blades and petioles are the main edible organs. Celery is a rich source of nutrients, such as dietary fiber, vitamins, proteins, carbohydrates, minerals and amino acids [5], along with phenolic acids, flavonoids (mainly quercetin, apigenin, chrysoeriol, luteolin and their glycosides), carotenoids, terpenoids and unsaturated fatty acids that exhibit biological activity and physiological functions in human beings [5,6,7]. The essential oil extracted from celery seeds ranged from 1.8 to 3.4% and contained approximately 20% selenine and 60% limonene [8].

Powdery mildew is among the most common fungal diseases that can cause substantial economic losses in a wide range of plants [9,10]. It is caused by different species of obligate parasitic fungi belonging to the order of Erysiphales (Ascomycota) [11,12,13,14] that favor warm and dry climates, although they require high relative humidity to spread [15]. On celery, powdery mildew caused by *Erysiphe heraclei* DC is one of the most destructive foliar diseases in greenhouse and field conditions [16]. The disease affects leave, petioles, umbels and fruits. White powdery patches, that get bigger as the disease progresses, appear on lower leaves, then spread to terminal growth. Infected leaves and petioles become brittle, distorted, and may eventually turn brown, shrivel and die, which could lead to serious and quality yield reductions. The spread of infection to umbels stops all flowers without seed formation [17,18,19].

The use of chemical fungicides is the primary means of controlling powdery mildew diseases [20,21]. However, these fungicides have their drawbacks as the development of resistance to them by pathogens is caused by the abusive use of fungicides. Moreover, environmental concerns regarding the negative effects of their overuse have serious implications for humans, animals, plants and other beneficial organisms [22,23] that have made alternative plant protection methods more favorable [24]. Recently, several promising approaches have been proposed as potential alternatives to fungicides; one is biological control using antagonistic microorganisms [25,26]. Biological control is currently accepted as a key practice in sustainable agriculture because it relies on managing natural resources and reducing the risks and potential negative effects of fungicides and has had remarkable success in controlling powdery mildew [27,28,29]. Hence, biological control strategies tend to provide a plant protection solution that is environmentally friendly, ecologically viable and has great potential to promote sustainable agriculture. Microorganisms in the genera *Bacillus*, *Pseudomonas*, *Serratia* and fungi which belong to the genera *Trichoderma* are the common biocontrol agents used in the disease control of fungal pathogens [30,31,32].

Biological control using *Trichoderma* spp. has been gaining interest worldwide [33]. Currently, more than 250 Trichoderma-based bio-fungicides are commercially available worldwide [34]. They are a type of soil fungi that are saprophytic, free-living and filamentous, often colonizing plants roots. These fungi have strong antagonistic effects against many phytopathogenic fungi [35]. By triggering multiple defense mechanisms, *Trichoderma* species show both resistance induction in plants and direct mycoparasitism of phytopathogenic fungi [36]. Synthesis and secretion of enzymes, secondary metabolites and antifungal compounds play a crucial part in all of these processes [37,38,39]. It was noted that *Trichoderma* isolates used as a seed treatment induced resistance to powdery mildew disease and dramatically improved plant growth [40].

It has been documented that many strains, which belong to the genus *Bacillus*, especially *B. subtilis*, are efficient in biologically controlling multiple plant pathogens [41,42]. Producing antibiotics by these bacteria is critical for disease inhibition [43]. Bacteria that are Gram-positive, in particular strains of *B. subtilis*, produce various antifungal and antibacterial antibiotics and fengycin family lipopeptides, which exhibit significant antifungal activities and growth suppression abilities of several plant pathogens [44,45]. Recently, several studies have confirmed the efficacy of *B. subtilis* against powdery mildew diseases under controlled or field conditions [46]. In general, members of the genus *Bacillus* are among the beneficial bacteria that are exploited as microbial pesticides, fungicides or fertilizers, as well as attaining biocontrol natures such as plant colonization, plant growth promotion, pathogen inhibition and activation of induced systemic resistance. Therefore, it is widely used in agriculture because of its environmental safety, good biological control efficacy for plant diseases and direct industrial production.

Plant defense mechanisms may involve changes in the enzymatic activity of several defense-related pathways. Defense-related enzymes such as peroxidase and polyphenol oxidase are mentioned as the plant induced systemic resistance (ISR) that correlates with disease control. These enzymes produced by the biosynthesis of plant metabolites, such as phenolic compounds, flavonoids, tannins and lignin [47,48], play an important role in plant stress tolerance, especially on fungal, bacterial and viral pathogens [49,50]. Many studies have indicated that the increase of defense-related enzymes activity due to greater accumulation of phenolic compounds can offer protection against plant diseases [25,26].

Therefore, this study was designed to investigate the potentiality of *T. album, T. harzianum*, *T. viride*, *B. subtilis, B. pumilus*, *B. megaterium*, *P. fluorescens* and *S. marcescens*, as well as the synthetic fungicide difenoconazole, to protect celery plants against powdery mildew. The study mainly focuses on the inhibitory effects of tested biocontrol agents on the germination of conidia of *E. heraclei* in vitro, in addition to their effect on growth and yield qualities, besides evaluating the biochemical changes that occur in the activities of defense-related enzymes, total phenols and photosynthetic pigments in response to the tested treatments in vivo conditions. The hypothesis tested was the possibility of using those biocontrol agents as eco-friendly alternatives instead of synthetic fungicide difenoconazole to protect celery plants against powdery mildew.

## 2. Results

### 2.1. In Vitro, Antifungal Activity of Biocontrol Agents against E. heraclei Conidia Germination and Germ Tube Length

In vitro, it was found that the antifungal activity of the tested biocontrol agents significantly reduced the conidia germination percentage and germ tube length of *E. heraclei* compared to the untreated control (Table 1). In particular, the fungicide difenoconazole and the bactericide were the most effective in reducing conidia germination by 97.1 and 88.2%, respectively, with a significant difference between them, followed by *B. pumilus* (84.1%), *S. marcescens* (81.1%), *B. megaterium* (79.9%) and *T. viride* (79.2%). While, *P. fluorescens* and *T. harzianum* reduced conidia germination by 76.6 and 73.8%, respectively. However, *T. album* ranked last (59.6%). Regarding germ tube length, the fungicide achieved the highest reduction (92.5%) with a significant difference, followed by *B. megaterium* (82.0%), *T. viride* (81.6%), *B. subtilis* (79.3%), *S. marcescens* (78.2%), *B. pumilus* (77.8%)*, T. harzianum* (77.3%) and *P. fluorescens* (74.7%) without significant differences between them, whereas, *T. album* was the least effective by 62.8% for the reduction in the germ tube length.

### 2.2. Investigation of the Potentiality of Biocontrol Agents against the Severity of Powdery Mildew and AUDPC

Data provided in Table 2 indicate that spraying celery plants with the tested biocontrol agents in the greenhouse significantly reduced the severity of powdery mildew, as well as the area under disease progress curve (AUDPC) at 7, 14, 21 and 28 days after application. In this regard, *B. subtilis* was the most efficient, followed by *B. pumilus*, *S. marcescens* and *B. megaterium*, with a significant difference, with reduction in disease severity by 80.1, 74.4, 73.2 and 70.5%, respectively, and a decrease in AUDPC by 285.3, 380.9, 396.7 and 431.8, over straight, compared to 1539.1 in untreated plants. The fungicide difenoconazole achieved maximum efficacy in terms of reducing the disease severity (84.7%) and the lowest AUDPC (219.3) compared to the other treatments.

### 2.3. In Vivo, the Efficiency of Biocontrol Agents in Suppressing Celery Powdery Mildew

Data presented in Table 3 reveal that all the applied biocontrol agents showed high efficiency in suppressing powdery mildew over control plants in both seasons, especially in plants treated with *B. subtilis* (77.9%), followed by *B. pumilus* (73.2%), with a significant difference, followed by *S. marcescens* (71.4%) and *B. megaterium* (67.6%), *T. viride* (63.5%) and *P. fluorescens* (61.2%). However, *T. album* (52.1%), followed by *T. harzianum* (58.1%) were the least effective. The fungicide difenoconazole had the superior efficiency in reducing disease severity (81.3%) compared to other treatments.

### 2.4. Growth and Yield Characteristics

**Figure 1:**
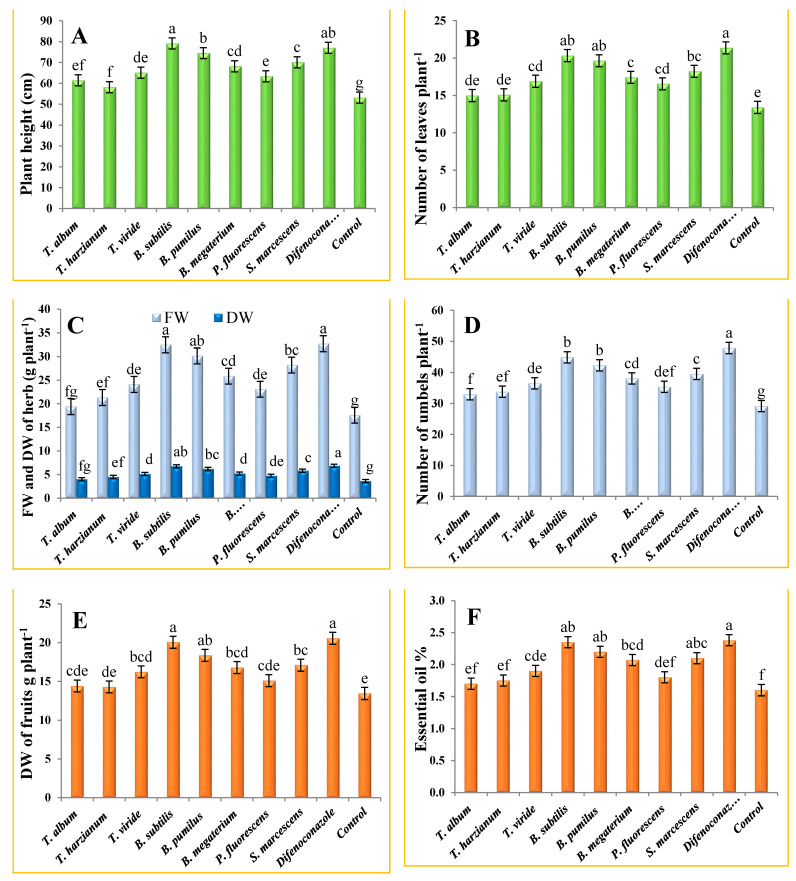
Effect of biocontrol agents and synthetic fungicide on (**A**) plant height; (**B**) number of leaves/plant; (**C**) fresh {FW} and dry weight {DW} of herb per plant; (**D**) number of umbels per plant; (**E**) dry weight of fruits per plant and (**F**) essential oil content of celery crop. Data are mean of two repeated trials during the growing seasons 2018/2019 and 2019/2020, each with three replicates. Different letters (a, b, c, etc.) on the columns show significant differences between the treatments according to Duncan’s multiple ranges test at 0.05 statistical level, ± bars indicate to standard errors of the mean.

All measured celery growth and yield characteristics improved significantly in response to the treatments compared to the untreated plants (Figure 1). *Bacillus subtilis,* followed by the fungicide difenoconazole, showed remarkable improvement in plant height compared to other treatments, recording 79.1 and 77.0 cm, respectively, without a significant difference (Figure 1A), followed by *B. pumilus*, *S. marcescens* and *B. megaterium*, by 74.5, 70.1 and 68.2 cm, respectively, while *T. harzianum* had the least effect (58.2 cm). Concerning the number of leaves/plant, the highest numbers were achieved in plants treated with the fungicide (21.4), *B. subtilis* (20.3), *B. pumilus* (19.6), *S. marcescens* (18.2) and *B. megaterium* (17.4), (Figure 1B). The lowest number of leaves/plant was recorded by *T. album* (14.9 leaves).

Moreover, all treatments significantly increased the fresh and dry weight of the herb/plant, and the best results were recorded by the fungicide, *B. subtilis* and *B. pumilus*, without significant differences, followed by *S. marcescens* and *B. megaterium*, recording 32.7, 32.5, 30.1, 28.2 and 25.9 g, respectively in FW and 6.9, 6.7, 6.2, 5.8 and 5.2 g, respectively in DW. The lowest values were recorded by *T. album* by 19.4 and 4.0 g in FW and DW, respectively (Figure 1C). Regarding the number of umbels/plant, the maximum value was recorded in plants treated with the fungicide (47.9 umbels/plant) and *B. subtilis* (44.9 umbels/plant), with a significant difference followed by *B. pumilus*, *S. marcescens*, *B. megaterium*, *T. viride* and *P. fluorescens*. The corresponding values were, 42.3, 39.5, 38.1, 36.5 and 35.4 umbels/plant, respectively, compared to the control plants (29.2 umbels/plant). However, the lowest values were recorded at 32.9 and 33.8 umbels/plant by *T. album* and *T. harzianum*, respectively (Figure 1D). Regarding the dry weight of fruits/plant, the greatest results were achieved by the fungicide (20.6 g), *B. subtilis* (20.1 g) and *B. pumilus* (18.4 g), without significant differences, followed by *S. marcescens* (17.1 g) and *B. megaterium* (16.8 g) compared to the control plants (13.5 g). The lowest value was recorded by *T. harzianum* (14.8 g) (Figure 1E).

Relating to the content of essential oil, all treatments significantly increased the essential oil percentage, especially the fungicide (2.4%), *B. subtilis* (2.4%), *B. pumilus* (2.2%) and *S. marcescens* (2.1%), without significant differences, followed by *B. megaterium* (2.1%) and *T. viride* (1.9%), compared to the untreated plants (1.6%). The lowest values were noted by *T. album*, *T. harzianum* and *P. fluorescens*, by 1.7, 1.8 and 1.8%, respectively (Figure 1F).

### 2.5. Chlorophyll and Carotenoid Content

**Figure 2:**
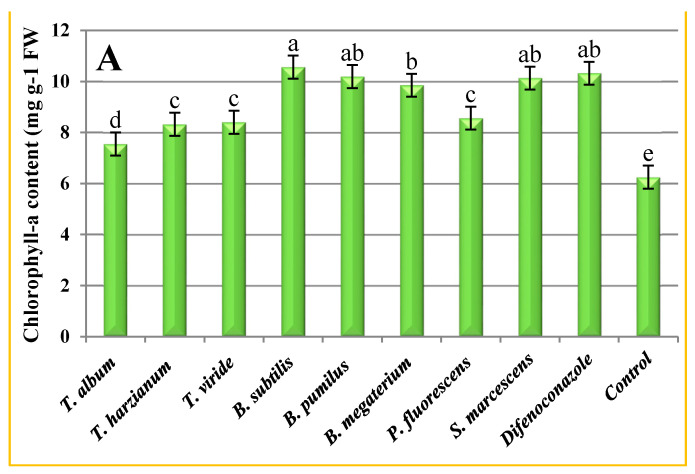

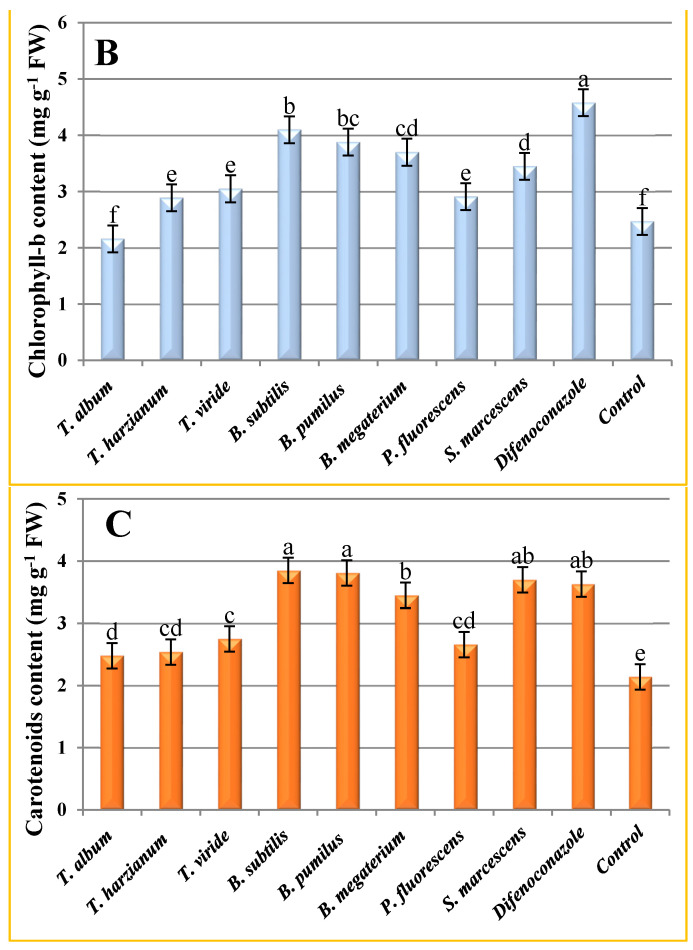
(**A**) Chlorophyll-a, (**B**) chlorophyll-b, and (**C**) carotenoids content (mg g^−1^ FW) of celery plant grown under different treatments of biocontrol agents and synthetic fungicide. Data are mean of two repeated trials during the growing seasons 2018/2019 and 2019/2020, each with three replicates. Different letters (a, b, c, etc.) on the columns show significant differences between the treatments according to Duncan’s multiple ranges test at 0.05 statistical level, ± bars indicate to standard errors of the mean.

The concentration of photosynthetic leaf pigments was significantly increased in celery leaves in response to the tested treatments (Figure 2). In particular, *B. subtilis* showed superiority in the containing of chlorophyll-a (Figure 2A), chlorophyll-b (Figure 2B) and carotenoids (Figure 2C), by (10.6, 4.3 and 3.9 mg·g^−1^ FW, respectively) followed by the fungicide difenoconazole (10.3, 4.2 and 3.8 mg g^−1^ FW, respectively), without significant differences between them except for the chlorophyll-b content, followed by *B. pumilus* (10.2, 3.9 and 3.8 mg g^−1^ FW, respectively), *S. marcescens* (10.1, 3.8 and 3.7 mg g^−1^ FW, respectively) and *B. megaterium* (9.9, 3.7 and 3.5 mg g^−1^ FW, respectively). Meanwhile, the lowest value was recorded by *T. album* (7.6, 2.6 and 2.5 mg g^−1^ FW, respectively) compared to the control plants (6.3, 2.5 and 2.1 mg g^−1^ FW, respectively).

### 2.6. Defense-Related Enzyme Activities and Total Phenol Content

Biocontrol agents as biotic inducers significantly increased the activity of peroxidase (PO) and polyphenol oxidase (PPO), as well as total phenol content (TPC) in powdery mildew-infected celery leaves (Table 4). The maximum levels of PO activity were recorded in plants treated with *B. subtilis* and *B. pumilus*, with a significant difference, 0.98 and 0.91 min^−1^ mg^−1^ protein, respectively, followed by *S. marcescens* and the fungicide difenoconazole, 0.89 and 0.85 min^−1^ mg^−1^ protein, respectively, compared to 0.42 min^−1^ mg^−1^ protein in the control plants. The lowest values were recorded by *T. album* and *T. harzianum*, 0.48 and 0.56 min^−1^ mg^−1^ protein, respectively. Regarding the activity of PPO, *B. pumilus* recorded superior activity, followed by *B. subtilis*, the fungicide and *S. marcescens*, by 0.45, 0.42, 0.40 and 0.38 min^−1^ mg^−1^ protein, respectively, compared to 0.11 min^−1^ mg^−1^ protein in the control plants. *T. album* was the least effective (0.14 min^−1^ mg^−1^ protein). Moreover, TPC significantly increased, especially by *B. subtilis*, *B. pumilus* and the fungicide, without significant differences compared to the control plants; the corresponding values of TPC were 50.1, 48.7, 47.6 and 26.1 mg g^−1^ FW, respectively. However, *T. album* was in the lowest order (29.5 mg g^−1^ FW).

## 3. Discussion

Due to the growing concern about health risks and environmental pollution from the use of chemical fungicides in agricultural production, it has become necessary to develop alternative strategies to control plant diseases and avoid the harmful effects of these fungicides. Hence, biological control offers an environmentally friendly alternative to the use of fungicides in suppressing plant diseases. The current study was designed to investigate the potentiality of certain biocontrol agents to protect celery plants against powdery mildew.

Under in vitro investigation, it was evident that conidia germination and germ tube length of *E. heraclei* were significantly reduced in response to the treatments. This finding is supported by other published papers [26,27]. The efficiency of biocontrol agents in reducing conidia germination may be attributed to their potent ability to synthesize and produce a wide variety of antibiotics and/or another direct inhibitory substances, such as hydrolytic enzymes, hydrogen cyanide and siderophores. These chemicals may degrade the cell wall of the spores or completely inhibit the enzymes required for the germination process [47,51]. Similarly, *Trichoderma* spp. have the ability to secrete several cell wall-degrading enzymes, i.e., endochitinase, chitobiosidase, n-acetyl-β-glucosaminidase and glucan 1, 3-β-glucosidase; these enzymes intensely inhibit spore germination and germ tube elongation [52]. In addition, *B. subtilis* and *Pseudomonas* spp. have been successful in controlling fungal and bacterial pathogens by inhibiting spore germination and disrupting germ tube and mycelial development [53]. The results also showed that difenoconazole had superior antifungal activity, recording a reduction of 97.13 and 92.45% in conidia germination and germ tube length, respectively. This action is probably due to its interference in the formation of conidia and haustoria [54].

In our study, all treatments achieved an obviously significant reduction in the severity of celery powdery mildew, as well as AUDPC. Among the tested biocontrol agents, *Bacillus* spp., especially *B. subtilis* and *B. pumilus*, were the most efficient. This efficiency may be attributed to the production of broad-spectrum antibiotics, such as surfactin, iturin and fengicin, which show significant antifungal activities and growth-suppressive capabilities for several plant pathogens [43,45]. Surfactin and iturin are able to modify bacterial surface hydrophobicity, and consequently, microbial adhesion to surfaces to mycelium [55]. In addition, iturin was found to act on sterols located in the cytoplasmic membrane of fungi [56]. *Bacillus subtilis* has the potential to produce cyclic peptide antibiotics, i.e., mycobacillinintrun-a, bacillomycin, mycosubtilin, fungistatin, subsoarinbacilysin and fongmycin-fiddaman [57], total proteins and hydrolytic enzymes, such as proteases, glucanases and chitinases, which degrades the mycelium of the pathogen [58], as well as siderophores, phytohormones, volatile compounds, lipopeptides and phytases that help stimulate plant growth and induce plant immune responses [41]. The results also indicate the efficacy of *Trichoderma* spp. against powdery mildew. This ability may be due to the competition of the nutrients, production of antibiotics, such as gliovirin, gliotoxin, viridin, pyrones and peptaibols [59], direct parasitism of pathogens and induction of plant systemic and localized resistance [60]. Furthermore, they have the ability to inhibit or degrade pectinases and other enzymes that are essential for plant pathogenic fungi [61] and secretion of cell wall lytic enzymes and secondary metabolites, such as endochitinase, chitobiosidase, n-acetyl-β-glucosaminidase and glucan 1, 3-β-glucosidase [62]. The results indicate the efficacy of *S. marcescens* in suppressing powdery mildew which could be attributed to its broad-spectrum, antifungal activity and production of lytic enzymes, HCN, IAA, siderophore and antibiotics [32,63]. However, *S. marcescens* is considered nowadays as an opportunistic pathogen for healthcare-associated infection and antimicrobial resistance [64]. On the other hand, it has been demonstrated that *P. fluorescens* can serve as a potential biocontrol agent due to its production of a number of secondary metabolites, such as antibiotics, lytic enzymes, i.e., β-1-3 glucanase, β-1-4 glucanase, chitinase and protease [65], siderophores and hydrogen cyanide [66]. The results also show that difenoconazole was the most efficient in reducing the disease severity and AUDPC. This action may be due to the interference of this fungicide in the biosynthesis of fungal sterols and the inhibition of the biosynthesis of ergosterol, which is necessary for the structure of the cell wall. Its absence leads to irrevocable damage to the cell wall, which leads to the death of the fungus [54].

In the current study, it was found that the measured growth and yield characteristics increased significantly in response to the treatments compared to the untreated plants in both seasons. Plant growth-promoting rhizobacteria (PGPR), such as *Bacillus* spp., *Pseudomonas* spp. and *Serratia* spp., are beneficial bacteria associated with many plant species and are commonly present in several environments [67,68]. These bacteria represent a promising alternative to plant growth-stimulating agents and plant health management [69,70], and enhance plant growth properties in direct and indirect ways; directly, by developing metabolites that provide soluble elements essential for plant nutrition, indirectly, they act as biological control agents by removing pathogens through the production of secondary metabolites [47,71]. PGPRs have the ability to directly promote plant growth through multiple mechanisms, such as biofilm formation [72], synthesis of phytohormones and sidérophores [73], nitrogen fixation [74], production of amino acids and vitamins [75], synthesis of some enzymes that modulate the level of plant hormones such as ACC deaminase [76], solubilization of inorganic phosphate and mineralization of organic phosphate, which makes phosphorus available for the plant [77], and the synthesis of growth-promoting compounds like indole acetic acid, gibberellic acid and cytokinins [48]. All of these mechanisms enhanced nutrient uptake, translocation and synthesis of photosynthetic assimilates resulting in increased plant growth and yield characters [78,79]. Furthermore, PGPRs can be used to increase plant yield under both stress and normal conditions by producing vitamins, antioxidants and several plant hormones [80,81] which can alleviate the damaging impacts of stress and improve yield production [82]. For example, it was found that plants grown under saline stress conditions and inoculated with *B. subtilis* had a higher plant growth, yield and nutrient uptake [83,84]. *Trichoderma* spp. plays an important role in improving plant growth and vigor by enhancing stress tolerance, active uptake of nutrients and bioremediation of contaminated rhizosphere and supplying plants with many secondary metabolites, enzymes and PR proteins [47,85], as well as producing plant growth promoters, such as gibberellic acid and biological control of some pathogenic fungi with increased plant growth [86,87].

Our results indicate that the concentration of photosynthetic pigments, chlorophyll a, chlorophyll b and carotenoids, was significantly increased in celery leaves in response to the treatments compared to the untreated plants. This finding is supported by previous studies [88,89]. The increase in photosynthetic leaf pigments may be attributed to the elevation of hormones and the enhancement of mineral absorption, i.e., (Fe and Me), which are essential for chlorophyll synthesis [90,91]. Furthermore, biocontrol agents may stimulate chlorophyll synthesis promoting the formation of pyridoxal enzymes, which play a role in α-amino levulinic synthetase as a primary compound in chlorophyll synthesis [58,62]. In addition, powdery mildew damages the structure and function of the mitochondria prior to chloroplasts, causing disruption of the inner and outer membranes of the affected plants [92]. Similarly, the reduction in chlorophyll content is mainly due to the intensification of powdery mildew infection and defoliation due to the disease [93]. The spread and penetration of pathogenic hyphae into host cells by haustoria is thought to destabilize the structural integrity, reducing chlorophyll pigments [94].

It is well known that defense-related enzymes play a crucial role in plant stress tolerance. In our investigation, all tested biocontrol agents as biotic inducers significantly increased the activity of PO and PPO, as well as TPC in powdery mildew-infected celery leaves. These results are consistent with previous studies that confirmed a positive relationship between plant resistance to powdery mildew, PO, PPO and TPC [25,26,95]. According to Safdarpour et al. [49], the activity of these enzymes has been associated with plant defense against fungal, bacterial and viral pathogens. He added that the importance of PPO’s activity in disease resistance probably related to its properties in the oxidation of phenolic compounds to quinines, which are often more toxic to pathogens than the original phenol. Polyphenol oxidase is also associated with lignification of cell walls, which contributes to defensive barriers to reinforce cell structure, thus playing a protective role in infected plants against pathogens [96]. Peroxidase is associated with many physiological activities within plant cells, such as regulation of plant cell elongation, phenol oxidation, polysaccharide cross linking, IAA oxidation, cross linking of extension monomers, oxidation of hydroxyl cinnamyl alcohols to free radical intermediates and wound healing [97], as well as the formation of lignin, a phenolic heteropolymer in the cell wall, which provides rigidity, strength, and resistance to chemical, physical and biological attacks in plants [98].

## 4. Material and Methods

### 4.1. Plant Material and Treatment Description

Celery seeds (*Apium graveolens* L.), Baladi cultivar used in this study, were obtained from the Horticultural Crops Technology Laboratory, National Research Center at Dokki, Giza, Egypt. Eight isolates (5 bacteria and 3 fungi), *Bacillus subtilis, B. pumilus*, *B. megaterium*, *Pseudomonas fluorescens*, *Serratia marcescens*, *Trichoderma album, T. harzianum*, *T. viride*, as well as the synthetic fungicide difenoconazole, were evaluated against the powdery mildew of celery. Both *B. subtilis* and *B. pumilus* were previously isolated from the surface of healthy celery plants and identified according to Zhang et al. [99] and Tabacchioni et al. [100]. While, the rest of the bacterial and fungal isolates were obtained from the Department of Microbiology, Soil, Water and Environment Res. Inst., ARC, Giza, Egypt. Fungicide difenoconazole, Dimethyl [1-((2-(2-chloro-4-(4-chlorophenoxy) phenyl)-4-methyl-1,3-dioxolan-2-yl) methyl)-1H-1,2,4-triazole)], is used at 50 mL /l00 L. (Syngenta Co., Basel, Switzerland). 

### 4.2. In Vitro, Evaluation of the Inhibitory Effect of Tested Treatments

#### 4.2.1. Preparation of Biocontrol Agent Inocula

Biocontrol agent fungal isolates used in this study were cultured separately on PDA media for 10 days, then cultures were flooded in 20 mL of sterile distilled water containing 0.02% Tween-80. The conidia were dislodged by gently scraping the colony surface with a spatula. The resulting suspension was shaken well and filtered through a sterilized cheesecloth to obtain a pure spore suspension, then spores were adjusted to 10^7^ spore mL^−1^ using a haemocytometer [101,102]. Biocontrol agent bacterial isolates were grown separately in a liquid nutrient broth in 250 mL flasks and kept on an orbital shaker at 150 rpm for 3–4 days. The pellets of each bacterium were suspended separately in tap water, then bacterial cells were adjusted to 10^9^ cell mL^−1^ [26].

#### 4.2.2. Preparation of Pathogenic Inoculum

The pathogenic inoculum of *E. heraclei* used in this study was prepared by collecting celery plants showing typical symptoms of powdery mildew. Pathogenicity was conducted on plants (45 days old) growing in a greenhouse [17], by shaking infected plants on healthy plants. Inoculated plants were kept in moist chambers (100% RH) at 25 ± 2 °C for 2 days, then moved into growth chambers at 22 ± 2 °C/ 16 ± 2 °C (day/night temperatures), 75% RH and 14 h photoperiod. Inoculated plants developed powdery mildew symptoms 12 to 15 days after inoculation. These symptoms and the fungus re-isolated from inoculated plants were morphologically identical to those originally observed on naturally infected plants. Profusely growing conidia were collected from these plants using a sterile camel hair brush and suspended in 100 mL of sterile distilled water containing 2 drops of Tween-20. The suspension was centrifuged at 3000 rpm for 5 min twice, in order to separate the conidia from conidiophores, then the conidia were adjusted to 5 × 10^5^ conidia mL^−1^ [103].

#### 4.2.3. Conidia Germination Assay

Conidia germination assay was performed by the detached leaf method technique described by [104]. Fresh celery leaves were washed in sterile distilled water. The spore suspension for each biocontrol agent was sprayed separately on the axial surface of the leaf using an atomizer. Leaves were inoculated with 3 drops of *E. heraclei* conidia suspension (5 × 10^5^ conidia mL^−1^) by spreading them uniformly over the leaf surface using a sterile camel hair brush. Control leaves were inoculated with the pathogenic conidia suspension only. The inoculated leaves were placed in 9-cm Petri-dishes having moist tissue paper (3 leaves/Petri-dish). Three Petri-dishes were used for each replicate, and 3 replicates for each treatment. Petri-dishes were incubated at 25 ± 1 °C for 48 h, then the leaves were examined for powdery conidia growth on the inoculated leaves. Conidia from the superficial growth were dusted on a water agar-coated slide using a hair brush (5 sweeps from each leaf for one slide), then incubated again for 48 h and examined under a light microscope (at 10×). Germinated conidia were counted by a haemocytometer, and its germ tube lengths (GTL) were measured (µ) by a micrometer. The percentage of conidia germination (CG) and the reduction in both CG and GTL were calculated using the following equations:

CG %=(Number of germinated conidia / Total number of germinated conidia)×100Reduction %=(Value in control – Value in treatment/ Value in control)×100 

### 4.3. In Vivo, Experimental Design

#### 4.3.1. Greenhouse Experiments

The experiments were carried out in the greenhouse at Fayoum Regional Research Station, Fayoum Governorate, Egypt during the growing season 2018/2019. Celery seeds were sown on 1 October in plastic pots (30 cm in diameter) previously filled with formalin- sterilized sandy loam soil (1:3 *w/w*), 12 kg in each pot containing 2–3 plants. The pots were completely randomized, with 5 pots per replicate, and 3 replicates per treatment. The plants were received in all plots all recommended by agricultural practices. Artificial inoculation with *E. heraclei* was performed by collecting fresh conidia from naturally infected celery plants having abundant sporulation and making a conidia suspension (5 × 10^5^ conidia mL^−1^). Healthy plants (45 days-old) were inoculated with the prepared conidia suspension using an atomizer. The inoculated plants were covered with polythene bags for 2 days at 25 ± 2 °C and 80–90% RH, then moved back to the growth chambers at 22 ± 2 °C/16 ± 2 °C (day/night temperatures), 75% RH and a 14 h photoperiod. Disease was assessed by estimating disease severity, which was performed at time 7, 14, 21 and 28 days after application.

#### 4.3.2. Field Experiments 

Two field experiments were conducted at the Experimental Farm, Faculty of Agriculture, Fayoum University, at Fayoum Governorate, Egypt (29°17′ N; 30°53′ E), to control celery powdery mildew during the growing seasons 2018/2019 and 2019/2020. The farm site was chosen as a result of previous observation of the disease on celery plants in it. Trials were arranged in a randomized, complete block design (RCBD) with 10 treatments, each containing 3 replicates. Celery seeds were sown on 1 October in plots (plot size: 2 × 3 m = 6 m^2^) with a distance of 30 cm between hills in the same row and 70 cm between rows at the rate of 3–7 seeds per hill, then the seedlings were thinned out to 2 in each hill 15 days after the date of germination. The plants in each plot were distributed into 3 rows, each plot containing 60 plants (30 hills) and each treatment consisted of 180 plants (90 hills). The plants were received in all plots all recommended by agricultural practices. The plants were left to natural powdery mildew infection, then sprayed with the tested treatments at a rate of 3 sprays with an interval of 2 weeks. The control plants were sprayed with water only (400 L ha^−1^). The disease severity was measured in each treatment 7 days after the last spray.

### 4.4. Measurements

#### 4.4.1. Disease Assessment 

Both artificially and naturally infected plants were carefully examined to estimate the severity of celery powdery mildew depending on the devised scale (0–5) according to Rathore and Rathore [105], where 0 = healthy plants, 1 = 1–20, 2 = 21–40, 3 = 41–60, 4 = 61–80, and 5 = 81–100% plant area covering with mildew. Observations on disease development were recorded from randomly selected twenty plants from each replicate and the disease severity was calculated as the following equation:

$$Disease\;severity\;\left(DS\right)\;\%=\left(\sum^{}\;\left(n\times v\right)/\;5\;N\right)\times 100$$

where; n = number of infected plants in each category, v = numerical values of each category, and N = total number of plants in the sample.

The efficiency of treatments in reducing disease severity was calculated using the following equation:

Efficiency %=(DS in control – DS in treatment/ DS in control)×100

The area under disease progress curve (AUDPC) was assessed at weekly intervals to compare different treatments on the severity of powdery mildew according to Pandey et al. [106] using the following equation:

AUDPC=D [1/2 (Y1+YK)+Y2+Y3+…YK−1]

where; D = days interval between two observations, Y1 = first disease severity, YK = last disease severity, and Y2, Y3, YK-1 = intermediate disease severity.

#### 4.4.2. Growth and Yield Attributes

Twenty plants were randomly selected from each experimental plot to measure their growth and yield characters. At 80 days-old, plant height, number of leaves/plant and fresh and dry weight of the herb/plant were measured. To measure the fresh weight of the herb, the stem was cut at 3 cm above the soil surface using a knife, the fresh tissue was immediately weighed on an electronic balance, while the dry weight of the herb was determined after the fresh herb was dried at 55 °C for 48 h. At physiological maturity (120 DAS), the number of umbels/plant (at full flowering stage), and dry weight of fruits/plant (at harvest stage) were measured. Yield increase was calculated using the following equation:

Yield increase %=(Yield in treatment – Yield in control/ Yield in control)×100

#### 4.4.3. Essential Oil Extraction

Approximately 100 g of dry, crushed celery fruits were extracted by hydro-distillation for 6 h using a Clevenger-type apparatus based on the method described by Phu et al. [107]. The obtained essential oil samples were dehydrated over an anhydrous sodium sulphate (Na_2_SO_4_) and then stored in dark sealed vials at 4 °C. The percentage of essential oil was calculated as the following equation:

(1) Essential oil %=(Observed volume of oil (mL)/ Weight of sample (g))×100 

### 4.5. Biochemical Assays

#### 4.5.1. Leaf Pigments

The leaf pigments of photosynthesis were measured according to the method described by Wellburn [108]. Approximately 200 mg of fresh celery leaves were homogenized in 50 mL of acetone (80% *v*/*v*) to estimate chlorophyll-a, chlorophyll-b and carotenoids (mg g^−1^ FW). The mixture was then centrifuged for 10 min at 10,000× *g* using a spectrophotometer (UV-160A, Shimadzu, Japan). The filtrated supernatant was read at wave lengths of 662, 645 and 470 nm. Equations used for calculation are presented below.

(2) Chlorophyll−a=11.75 A662 – 2.350 A645Chlorophyll−b=18.61 A645 – 3.960 A662Carotene=1000 A470 – 2.270 Chl. a – 81.4 Chl. b/227Chlorophyll or carotene (mg/g FW)=Chlorophyll or carotene content−Volume of acetone/1000−Weight of sample (g)

#### 4.5.2. Peroxidase (PO) Activity

The activity of the PO was assayed by the method described by Thimmaiah [109]. One gram of fresh leaves was macerated in previously chilled mortar containing 10 mL of 0.1 M phosphate buffer (pH 6). The homogenate was strained through 2 folds of muslin cloth and centrifuged at 16,000× *g* for 20 min at 4 °C. The supernatant was used as an enzyme source. 1 mL 0-dianisidine, 0.5 mL H_2_O_2_, 1 mL of phosphate buffer and 2.4 mL of distilled water were pipetted in test tubes. The blank was prepared by excluding H_2_O_2_ and adding an additional volume of water in the place of H_2_O_2_. The reaction was started by adding 0.2 mL of an enzyme extract and incubating at 30 °C for 5 min and stopped by adding 1 mL of 2NH_2_SO_4_. The absorbance was measured at 430 nm against a reagent blank. The unit of enzyme was defined as absorbance/min/mg protein.

#### 4.5.3. Polyphenol Oxidase (PPO) Activity

The activity of the PPO was determined by the method of Mayer et al. [110]. One gram of fresh leaves was homogenized in 2 mL 0.1 M of a sodium phosphate buffer at pH 6.5 and centrifuged at 16,000× *g* for 15 min at 4 °C. The supernatant was used as an enzyme source. The reaction mixture consisted of 0.2 mL of enzyme extract (supernatant) and 1.5 mL of 0.1 M sodium phosphate buffer (pH 6.5). To start the reaction, 0.2 mL of 0.1 M catechol was added and the change in absorbance was recorded at 30 s intervals for up to 3 min at 495 nm. The unit of enzyme was defined as absorbance/min/mg protein.

#### 4.5.4. Total Phenol Content (TPC)

The content of total phenols was determined by the method of Fu et al. [111]. After extraction, an aliquot of 0.2 mL was taken and mixed with 1.0 mL of the Folin-Ciocalteu reagent 1:10 and 0.8 mL of 7.5% NaCO_3_, followed by stirring. After 30 min of rest in the dark, the spectrophotometer reading at 760 nm was performed, using gallic acid as a standard. The phenol content was expressed as mg/ g FW (Fresh Weight).

### 4.6. Statistical Analysis

The data were subjected to statistical analysis by ANOVA, using WASP software (Web Agriculture Stat Package). The values presented are the means of all measurements. A combined analysis was used for the data obtained from the two seasons of the current study, as well as Duncan’s range test for comparing the significant differences between different treatments at *p* ≤ 0.05, as outlined by Gomez and Gomez [112].

## 5. Conclusions

Our investigative study indicated that the application of biocontrol agents, such as *Bacillus* spp., *Trichoderma* spp., *S. marcescens* and *P. fluorescens*, significantly reduced the germination of conidia and germ tube length of *E. heraclei*, in vitro compared to the control. It also showed superior efficiency in reducing the severity of celery powdery mildew, as well as the area under disease progress curve (AUDPC), after application in the greenhouse. In the field, all the applied biocontrol agents showed a high efficiency in suppressing powdery mildew on celery plants, with a significant improvement in growth and yield characteristics, and a significant increase of leaf pigments concentration, the activities of the defense-related enzymes such as peroxidase (PO) and polyphenol oxidase (PPO), and total phenol content (TPC). In conclusion, the results demonstrated the possibility of using those biocontrol agents as eco-friendly alternatives to protect celery plants against powdery mildew.

## Figures and Tables

**Table 1:** In vitro, the inhibitory effect of biocontrol agents on conidia germination percentage and germ tube length of *E. heraclei* evaluated after 48 h of treatment and incubation at 25 ± 1 °C.

Treatments	Concentration	Conidia Germination (CG)		Germ Tube Length (GTL)	
		CG%	* Reduction%	GTL (µ)	* Reduction%
*T. album*	10^7^ spore mL^−1^	25.14 ± 2.52 ^b^	59.57	15.32 ± 2.15 ^b^	62.84
*T. harzianum*	10^7^ spore mL^−1^	16.29 ± 2.1 ^c^	73.80	9.36 ± 1.5 ^c^	77.29
*T. viride*	10^7^ spore mL^−1^	12.95 ± 0.88 ^cde^	79.17	7.59 ± 0.56 ^c^	81.59
*B. subtilis*	10^9^ cfu mL^−1^	7.31 ± 0.61 ^f^	88.24	8.55 ± 0.79 ^c^	79.26
*B. pumilus*	10^9^ cfu mL^−1^	9.88 ± 1.22 ^ef^	84.11	9.16 ± 1.63 ^c^	77.78
*B. megaterium*	10^9^ cfu mL^−1^	12.48 ± 0.65 ^cde^	79.92	7.41 ± 0.36 ^c^	82.02
*P. fluorescens*	10^9^ cfu mL^−1^	14.53 ± 1.77 ^cd^	76.63	10.44 ± 2.68 ^c^	74.67
*S. marcescens*	10^9^ cfu mL^−1^	11.77 ± 1.12 ^de^	81.07	8.97 ± 0.46 ^c^	78.24
Difenoconazole	0.5 mL L^−1^	1.78 ± 0.26 ^g^	97.13	3.11 ± 0.19 ^d^	92.45
Control	–	62.18 ± 5.71 ^a^	–	41.23 ± 2.89 ^a^	–

CG = conidia germination, and GTL = germ tube length. * Values are calculated according to the control value. Values followed by the same letter within the same column are not significantly different at a *p* ≤ 0.05 statistical level, according to Duncan’s multiple range test.

**Table 2:** Effect of tested biocontrol agents on the severity of celery powdery mildew (*E. heraclei*) and the area under disease progress curve (AUDPC) in greenhouse conditions.

Treatments	Disease Severity%				* Efficiency%	AUDPC
	Days after Starting the Application					
	7 Days	14 Days	21 Days	28 Days		
*T. album*	18.22 ± 1.54 ^b^	25 ± 0.96 ^b^	33.15 ± 1.87 ^a^	50.38 ± 2.68 ^a^	55.47	647.15 ± 28.73 ^b^
*T. harzianum*	15.76 ± 2.09 ^bc^	22.9 ± 2.4 ^bc^	31 ± 1.4 ^b^	45.89 ± 1.47 ^c^	60.42	593.07 ± 25.21 ^c^
*T. viride*	14.32 ± 0.97 ^c^	19.64 ± 0.92 ^de^	25.35 ± 1.73 ^cd^	42.54 ± 1.92 ^c^	65.11	513.94 ± 27.11 ^d^
*B. subtilis*	7.37 ± 0.68 ^ef^	10.83 ± 1.57 ^g^	15.22 ± 1.75 ^f^	22.05 ± 1.89 ^g^	80.13	285.32 ± 11.36 ^g^
*B. pumilus*	7.52 ± 0.71 ^ef^	13.4 ± 1.48 ^fg^	20.58 ± 1.78 ^e^	33.35 ± 2.06 ^e^	74.36	380.91 ± 15.15 ^f^
*B. megaterium*	11.47 ± 1.2 ^d^	18.03 ± 1.74 ^e^	23.19 ± 1.1 ^de^	29.46 ± 1.57 ^f^	70.49	431.79 ± 23.16 ^e^
*P. fluorescens*	15.31 ± 2.03 ^c^	20.98 ± 1.67 ^cd^	27.44 ± 1.27 ^c^	38.63 ± 2.6 ^d^	63.85	527.73 ± 33.2 ^d^
*S. marcescens*	9.55 ± 1.32 ^de^	13.75 ± 0.7 ^f^	21.38 ± 0.91 ^e^	33.52 ± 3.56 ^e^	73.21	396.65 ± 24.92 ^f^
Difenoconazole	5.58 ± 1.33 ^f^	7.87 ± 0.51 ^h^	12.5 ± 1.39 ^f^	16.33 ± 1.15 ^h^	84.67	219.27 ± 19.37 ^h^
Control	37.49 ± 3.23 ^a^	64.32 ± 2.65 ^a^	88.13 ± 2.96 ^b^	97.35 ± 1.19 ^b^	–	1539.10 ± 95.75 ^a^

AUDPC = area under disease progress curve. * Values are calculated according to the control value. Values followed by the same letter within the same column are not significantly different at a *p* ≤ 0.05 statistical level, according to Duncan’s multiple range test.

**Table 3:** Effect of tested biocontrol agents on the severity of celery powdery mildew during the growing seasons 2018/2019 and 2019/2020 in field conditions.

Treatments	Disease Severity%			* Efficiency%
	Growing Season		Mean	
	2018/2019	2019/2020		
*T. album*	29.42 ± 1.84 ^b^	32.2 ± 1.13 ^b^	30.81	52.13
*T. harzianum*	28.91 ± 1.32 ^b^	25.09 ± 1.94 ^cd^	27.00	58.05
*T. viride*	21.58 ± 1.12 ^cd^	25.44 ± 1.66 ^cd^	23.51	63.47
*B. subtilis*	16.4 ± 0.97 ^f^	12.04 ± 0.96 ^f^	14.22	77.90
*B. pumilus*	18.33 ± 1.21 ^ef^	16.23 ± 1.85 ^e^	17.28	73.15
*B. megaterium*	19.56 ± 1.1 ^de^	22.2 ± 1.39 ^d^	20.88	67.56
*P. fluorescens*	22.64 ± 2.83 ^c^	27.32 ± 0.76 ^c^	24.98	61.19
*S. marcescens*	19.5 ± 1.09 ^de^	17.38 ± 4.27 ^e^	18.44	71.35
Difenoconazole	11.48 ± 0.92 ^g^	12.62 ± 0.99 ^f^	12.05	81.28
Control	61.34 ± 2.36 ^a^	67.4 ± 3.14 ^a^	64.37	–

* Values are calculated according to the control value. Values followed by the same letter within the same column are not significantly different at a *p* ≤ 0.05 statistical level, according to Duncan’s multiple range test.

**Table 4:** Peroxidase and polyphenol oxidase activities and total phenol content in powdery mildew-infected celery leaves in response to biocontrol agents and synthetic fungicide during the growing seasons 2018/2019 and 2019/2020 under field conditions.

Treatments	PO Activity (min^−1^ mg^−1^ Protein)	PPO Activity (min^−1^ mg^−1^ Protein)	TPC (mg g^−1^ FW)
*T. album*	0.48 ± 0.03 ^g^	0.14 ± 0.02 ^g^	29.47 ± 2.29 ^d^
*T. harzianum*	0.56 ± 0.02 ^f^	0.21 ± 0.01 ^ef^	35.22 ± 1.6 ^c^
*T. viride*	0.72 ± 0.02 ^d^	0.24 ± 0.01 ^e^	38.16 ± 2.68 ^bc^
*B. subtilis*	0.98 ± 0.06 ^a^	0.42 ± 0.02 ^ab^	50.12 ± 1.89 ^a^
*B. pumilus*	0.91 ± 0.03 ^b^	0.45 ± 0.02 ^a^	48.65 ± 1.3 ^a^
*B. megaterium*	0.76 ± 0.02 ^d^	0.29 ± 0.02 ^d^	40.69 ± 2.87 ^b^
*P. fluorescens*	0.64 ± 0.05 ^e^	0.20 ± 0.02 ^f^	34.53 ± 1.74 ^c^
*S. marcescens*	0.89 ± 0.02 ^bc^	0.38 ± 0.02 ^c^	41.90 ± 1.15 ^b^
Difenoconazole	0.85 ± 0.03 ^c^	0.40 ± 0.02 ^bc^	47.58 ± 2.91 ^a^
Control	0.42 ± 0.02 ^h^	0.11 ± 0.02 ^h^	26.13 ± 1.28 ^d^

PO = peroxidase, PPO = polyphenol oxidase, and TPC = total phenol content. Values followed by the same letter within the same column are not significantly different at a *p* ≤ 0.05 statistical level, according to Duncan’s multiple range test.

## Data Availability

All data is presented within the article.
